# Dichlorido[(*Z*)-4-(2,6-diisopropyl­anilino)pent-3-en-2-one]dimethyl­tin(IV)

**DOI:** 10.1107/S1600536809055810

**Published:** 2010-01-09

**Authors:** Ciprian Raţ, Carmen Comşa, Cristian Silvestru

**Affiliations:** a Universitatea Babeş-Bolyai, Facultatea de Chimie şi Inginerie Chimicã, 11 Arany Janos, 400028 Cluj-Napoca, Romania

## Abstract

In the crystal structure of the title compound, [Sn(CH_3_)_2_Cl_2_(C_17_H_25_NO)], the Sn atom adopts a trigonal-bipyramidal geometry with the O and one Cl atom in axial positions. A weak intra­molecular N—H⋯O hydrogen bond occurs. The crystal structure displays weak inter­molecular C—H⋯Cl inter­actions.

## Related literature

For dichloridodiorganotin(IV) complexes, see: Cunningham *et al.* (2004 ▶); Curnow *et al.* (2006 ▶); Ianelli *et al.* (1993 ▶); Mahadevan *et al.* (1982 ▶); Ng (1996 ▶); Papadaki *et al.* (2008 ▶); Tian *et al.* (2006 ▶); Valle *et al.* (1982 ▶).
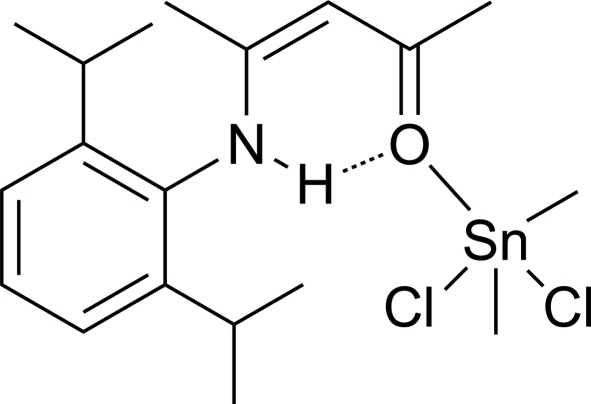


## Experimental

### 

#### Crystal data

[Sn(CH_3_)_2_Cl_2_(C_17_H_25_NO)]
*M* *_r_* = 479.04
Triclinic, 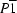

*a* = 8.504 (4) Å
*b* = 10.212 (4) Å
*c* = 14.507 (6) Å
α = 71.070 (7)°
β = 83.300 (8)°
γ = 76.984 (7)°
*V* = 1159.8 (9) Å^3^
*Z* = 2
Mo *K*α radiation
μ = 1.34 mm^−1^
*T* = 297 K
0.36 × 0.35 × 0.33 mm

#### Data collection

Bruker SMART APEX CCD area-detector diffractometer
Absorption correction: multi-scan (*SADABS*; Bruker, 2000 ▶) *T*_min_ = 0.626, *T*_max_ = 0.645
8415 measured reflections
4044 independent reflections
3433 reflections with *I* > 2σ(*I*)
*R*_int_ = 0.028

#### Refinement

*R*[*F* ^2^ > 2σ(*F* ^2^)] = 0.046
*wR*(*F* ^2^) = 0.104
*S* = 1.06
4044 reflections
229 parameters
1 restraint
H atoms treated by a mixture of independent and constrained refinement
Δρ_max_ = 0.57 e Å^−3^
Δρ_min_ = −0.41 e Å^−3^

### 

Data collection: *SMART* (Bruker, 2000 ▶); cell refinement: *SAINT-Plus* (Bruker, 2001 ▶); data reduction: *SAINT-Plus*; program(s) used to solve structure: *SHELXS97* (Sheldrick, 2008 ▶); program(s) used to refine structure: *SHELXL97* (Sheldrick, 2008 ▶); molecular graphics: *DIAMOND* (Brandenburg, 2009 ▶); software used to prepare material for publication: *PLATON* (Spek, 2009 ▶).

## Supplementary Material

Crystal structure: contains datablocks I, global. DOI: 10.1107/S1600536809055810/nc2173sup1.cif

Structure factors: contains datablocks I. DOI: 10.1107/S1600536809055810/nc2173Isup2.hkl

Additional supplementary materials:  crystallographic information; 3D view; checkCIF report

## Figures and Tables

**Table:** 

C18—Sn1	2.095 (5)
C19—Sn1	2.105 (5)
Cl1—Sn1	2.3478 (16)
Cl2—Sn1	2.4644 (17)
O1—Sn1	2.375 (3)

**Table:** 

C18—Sn1—C19	142.9 (3)
Cl1—Sn1—O1	81.74 (9)
C18—Sn1—Cl2	94.04 (15)
C19—Sn1—Cl2	94.78 (18)
Cl1—Sn1—Cl2	95.82 (6)
O1—Sn1—Cl2	176.81 (8)

**Table 2:** Hydrogen-bond geometry (Å, °)

*D*—H⋯*A*	*D*—H	H⋯*A*	*D*⋯*A*	*D*—H⋯*A*
N1—H1⋯O1	0.83 (3)	2.03 (4)	2.662 (5)	133 (4)
C8^i^—H8^i^⋯Cl1	0.93	2.91	3.695 (4)	143
C17^ii^—H17*B*^ii^⋯Cl2	0.96	2.89	3.783 (6)	155
C19^iii^—H19*C*^iii^⋯Cl2	0.96	2.94	3.700 (6)	137

Symmetry codes: (i) 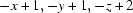; (ii) 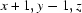; (iii) 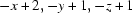.

**Fig. 1.**
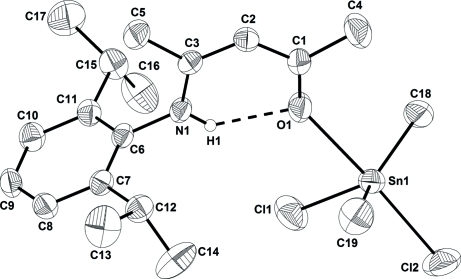
Crystal structure of the title compound with labelling and displacement ellipsoids drawn at 30% probability level. Hydrogen atoms, except that bonded to nitrogen, were omitted for clarity. Intramolecular hydrogen bonding is shown as a dashed line.

## supplementary crystallographic information

### Comment

In our attempts to prepare compounds of type Me_2_R'_2_Sn, where R' = ketinimate ligand, starting from RLi and Me_2_SnCl_2_, accidental hydrolysis of the lithium derivative lead to the formation of the title complex. A rational preparation of the complex was acomplished later starting from R'H and Me_2_SnCl_2_.

In the structure of the title compound the geometry around the tin can be described as distorted trigonal bipyramidal, with the Cl(2) and O(1) atoms occupying the axial positions (Fig. 1). The equatorial plane is formed by the two methyl carbon atoms C(18) and C(19) and the Cl(1) atom.

A weak intramolecular hydrogen bond exist between the hydrogen atom bonded to nitrogen and the oxygen atom (Table 2). There are additional weak C–H···Cl interactions (Table 2).

### Experimental

A solution of Me_2_SnCl_2_ in Et_2_O (0.84 g, 3.82 mmol) was added to a stirred solution of (*Z*)-4-[(2,6-diisopropylphenyl)amino]pent-3-en-2-one (1 g, 3.85 mmol) in 50 ml Et_2_O resulting in a clear red-brown solution. The reaction mixture was stirred for 24 h and than the solvent was removed under reduced presure to give the title compound as a white-yellow powder. Crystals were obtained by slow diffusion of hexane into a dichloromethane solution of the title compound. Yield: 0.6 g (33%). mp = 124–125 °C.

^1^H NMR (CDCl_3_, 300 MHz): δ 1.15 [d, 6H_A_, –CH(C*H*_3_)_2_, ^3^J(H,H) = 6.8 Hz], 1.19 [s, 6H, SnC*H*_3_, ^2^J(^117^Sn,H) = 75.3, ^2^J(^119^Sn,H) = 78.7 Hz], 1.20 [d, 6H_B_, –CH(C*H*_3_)_2_, ^3^J(H,H) = 6.9 Hz], 1.67 [s, 3H, C*H*_3_C(N)], 2.11 [s, 3H, C*H*_3_C(O)], 2.92 [sept, 2H, –C*H*(CH_3_)_2_, ^3^J(H,H) = 6.9 Hz], 5.22 [s, 1H, –C*H*-], 7.18 [d, 2H, *H*_8,10_, ^3^J(H,H) = 7.7 Hz], 7.32 [t, 1H, *H*_9_, ^3^J(H,H) = 7.7 Hz], 11.83 [s, 1H, –N*H*-].

^13^C NMR (CDCl_3_, 75.5 MHz): δ 10.73 [s, Sn*C*H_3_, ^1^J(^117^Sn,*C*) = 587.1, ^1^J(^119^Sn,*C*) = 614.4 Hz], 19.49 [s, *C*H_3_C(N)], 22.57 [s, –CH(*C*H_3_)_2_, (B)], 24.45 [s, –CH(*C*H_3_)_2_, (A)], 28.01 [s, -*C*H(CH_3_)_2_], 28.43 [s, *C*H_3_C(O)], 96.31 [s, -*C*H–], 123.68 [s, *C*_8,10_], 128.81 [s, *C*_9_], 132.27 [s, *C*_6_], 145.53 [s, *C*_7,11_], 166.74 [s, CH_3_*C*(N)], 193.59 [s, CH_3_*C*(O)].

^119^Sn NMR (CDCl_3_, 111.9 MHz): δ -1.33.

### Refinement

The C-H H atoms were placed in calculated positions (methyl H atoms allowed to rotate but not to tip) with *U*_iso_(H) = 1.2*U*_eq_(C) (1.5 for methyl H atoms). The N-H H atom was located in a difference map and its position was refined with istotropic displacement parameters with a restrained N–H distance of 0.86 Å.

### Crystal data

**Table:**

[Sn(CH_3_)_2_Cl_2_(C_17_H_25_NO)]	*Z* = 2
*M**_r_* = 479.04	*F*(000) = 488
Triclinic, *P*1	*D*_x_ = 1.372 Mg m^−^^3^
Hall symbol: -P 1	Melting point = 397–398 K
*a* = 8.504 (4) Å	Mo *K*α radiation, λ = 0.71073 Å
*b* = 10.212 (4) Å	Cell parameters from 2254 reflections
*c* = 14.507 (6) Å	θ = 2.2–20.4°
α = 71.070 (7)°	µ = 1.34 mm^−^^1^
β = 83.300 (8)°	*T* = 297 K
γ = 76.984 (7)°	Block, colourless
*V* = 1159.8 (9) Å^3^	0.36 × 0.35 × 0.33 mm

### Data collection

**Table:**

Bruker SMART APEX CCD area-detector diffractometer	4044 independent reflections
Radiation source: fine-focus sealed tube	3433 reflections with *I* > 2σ(*I*)
graphite	*R*_int_ = 0.028
φ and ω scans	θ_max_ = 25°, θ_min_ = 2.2°
Absorption correction: multi-scan (*SADABS*; Bruker, 2000)	*h* = −10→10
*T*_min_ = 0.626, *T*_max_ = 0.645	*k* = −12→12
8415 measured reflections	*l* = −17→17

### Refinement

**Table:**

Refinement on *F*^2^	Primary atom site location: structure-invariant direct methods
Least-squares matrix: full	Secondary atom site location: difference Fourier map
*R*[*F*^2^ > 2σ(*F*^2^)] = 0.046	Hydrogen site location: inferred from neighbouring sites
*wR*(*F*^2^) = 0.104	H atoms treated by a mixture of independent and constrained refinement
*S* = 1.06	*w* = 1/[σ^2^(*F*_o_^2^) + (0.045*P*)^2^ + 0.2662*P*] where *P* = (*F*_o_^2^ + 2*F*_c_^2^)/3
4044 reflections	(Δ/σ)_max_ = 0.001
229 parameters	Δρ_max_ = 0.57 e Å^−^^3^
1 restraint	Δρ_min_ = −0.41 e Å^−^^3^

### Special details

**Table:**

Geometry. All e.s.d.'s (except the e.s.d. in the dihedral angle between two l.s. planes) are estimated using the full covariance matrix. The cell e.s.d.'s are taken into account individually in the estimation of e.s.d.'s in distances, angles and torsion angles; correlations between e.s.d.'s in cell parameters are only used when they are defined by crystal symmetry. An approximate (isotropic) treatment of cell e.s.d.'s is used for estimating e.s.d.'s involving l.s. planes.
Refinement. Refinement of *F*^2^ against ALL reflections. The weighted *R*-factor *wR* and goodness of fit *S* are based on *F*^2^, conventional *R*-factors *R* are based on *F*, with *F* set to zero for negative *F*^2^. The threshold expression of *F*^2^ > σ(*F*^2^) is used only for calculating *R*-factors(gt) *etc*. and is not relevant to the choice of reflections for refinement. *R*-factors based on *F*^2^ are statistically about twice as large as those based on *F*, and *R*- factors based on ALL data will be even larger.

### Fractional atomic coordinates and isotropic or equivalent isotropic displacement parameters (Å^2^)

**Table:**

	*x*	*y*	*z*	*U*_iso_*/*U*_eq_	
C1	0.6984 (5)	0.9135 (5)	0.5930 (3)	0.0605 (11)	
C2	0.6594 (5)	1.0064 (4)	0.6472 (3)	0.0541 (10)	
H2	0.6908	1.0931	0.6209	0.065*	
C3	0.5787 (5)	0.9812 (4)	0.7360 (3)	0.0494 (10)	
C4	0.7852 (8)	0.9572 (6)	0.4950 (4)	0.101 (2)	
H4A	0.8739	0.8825	0.4899	0.152*	
H4B	0.8252	1.0408	0.4879	0.152*	
H4C	0.7119	0.9762	0.4446	0.152*	
C5	0.5455 (7)	1.0906 (5)	0.7874 (4)	0.0781 (15)	
H5A	0.4309	1.1209	0.7959	0.117*	
H5B	0.592	1.17	0.7494	0.117*	
H5C	0.5922	1.0511	0.8501	0.117*	
C6	0.4417 (5)	0.8299 (4)	0.8734 (3)	0.0486 (10)	
C7	0.5269 (5)	0.7605 (4)	0.9578 (3)	0.0527 (10)	
C8	0.4401 (6)	0.7330 (5)	1.0457 (3)	0.0603 (11)	
H8	0.494	0.685	1.103	0.072*	
C9	0.2759 (6)	0.7751 (5)	1.0501 (3)	0.0641 (12)	
H9	0.2195	0.7569	1.1103	0.077*	
C10	0.1943 (5)	0.8433 (5)	0.9674 (3)	0.0648 (12)	
H10	0.0826	0.872	0.9716	0.078*	
C11	0.2755 (5)	0.8706 (5)	0.8765 (3)	0.0565 (11)	
C12	0.7095 (5)	0.7131 (5)	0.9532 (4)	0.0650 (12)	
H12	0.7507	0.774	0.8923	0.078*	
C13	0.7902 (7)	0.7273 (8)	1.0355 (5)	0.109 (2)	
H13A	0.7611	0.6615	1.0958	0.163*	
H13B	0.7556	0.8218	1.0392	0.163*	
H13C	0.9053	0.7078	1.0239	0.163*	
C14	0.7528 (8)	0.5650 (7)	0.9482 (6)	0.120 (2)	
H14A	0.7234	0.5013	1.0095	0.18*	
H14B	0.867	0.5408	0.9348	0.18*	
H14C	0.6957	0.558	0.8972	0.18*	
C15	0.1811 (6)	0.9386 (6)	0.7843 (4)	0.0727 (14)	
H15	0.2549	0.979	0.7309	0.087*	
C16	0.1201 (8)	0.8259 (8)	0.7587 (4)	0.112 (2)	
H16A	0.2096	0.752	0.7532	0.169*	
H16B	0.068	0.8675	0.6977	0.169*	
H16C	0.0444	0.7874	0.809	0.169*	
C17	0.0427 (8)	1.0556 (8)	0.7938 (5)	0.132 (3)	
H17A	−0.0347	1.0172	0.843	0.198*	
H17B	−0.0081	1.1	0.7326	0.198*	
H17C	0.0825	1.1242	0.8119	0.198*	
C18	0.6696 (7)	0.6652 (5)	0.4531 (3)	0.0799 (15)	
H18A	0.5909	0.7496	0.4515	0.12*	
H18B	0.6202	0.6001	0.4372	0.12*	
H18C	0.7568	0.6886	0.4065	0.12*	
C19	0.9525 (7)	0.5569 (7)	0.6771 (4)	0.1025 (19)	
H19A	0.9978	0.4592	0.7077	0.154*	
H19B	0.9138	0.6023	0.7262	0.154*	
H19C	1.0339	0.6022	0.6356	0.154*	
Cl1	0.5420 (2)	0.51095 (17)	0.70215 (11)	0.1040 (5)	
Cl2	0.8568 (2)	0.33755 (15)	0.57138 (13)	0.1102 (6)	
H1	0.547 (5)	0.801 (3)	0.752 (3)	0.055 (13)*	
N1	0.5289 (4)	0.8616 (4)	0.7806 (3)	0.0513 (8)	
O1	0.6627 (4)	0.7925 (3)	0.6232 (2)	0.0761 (10)	
Sn1	0.75936 (4)	0.57249 (3)	0.59303 (2)	0.06287 (15)	

### Atomic displacement parameters (Å^2^)

**Table:**

	*U*^11^	*U*^22^	*U*^33^	*U*^12^	*U*^13^	*U*^23^
C1	0.064 (3)	0.058 (3)	0.056 (3)	−0.011 (2)	0.010 (2)	−0.019 (2)
C2	0.060 (3)	0.047 (2)	0.056 (3)	−0.018 (2)	0.003 (2)	−0.014 (2)
C3	0.048 (2)	0.046 (2)	0.057 (3)	−0.0075 (19)	−0.0061 (19)	−0.019 (2)
C4	0.131 (5)	0.096 (4)	0.077 (4)	−0.048 (4)	0.042 (3)	−0.027 (3)
C5	0.111 (4)	0.056 (3)	0.078 (3)	−0.024 (3)	0.013 (3)	−0.036 (3)
C6	0.052 (2)	0.046 (2)	0.053 (2)	−0.0107 (19)	0.004 (2)	−0.024 (2)
C7	0.056 (3)	0.050 (2)	0.057 (3)	−0.011 (2)	−0.002 (2)	−0.023 (2)
C8	0.072 (3)	0.059 (3)	0.050 (3)	−0.015 (2)	−0.001 (2)	−0.016 (2)
C9	0.073 (3)	0.069 (3)	0.052 (3)	−0.019 (3)	0.017 (2)	−0.024 (2)
C10	0.052 (3)	0.074 (3)	0.067 (3)	−0.014 (2)	0.004 (2)	−0.023 (3)
C11	0.060 (3)	0.059 (3)	0.054 (3)	−0.014 (2)	0.002 (2)	−0.022 (2)
C12	0.055 (3)	0.065 (3)	0.074 (3)	−0.008 (2)	−0.004 (2)	−0.022 (2)
C13	0.069 (4)	0.152 (6)	0.125 (5)	−0.024 (4)	−0.020 (4)	−0.062 (5)
C14	0.087 (4)	0.093 (5)	0.188 (7)	0.020 (4)	−0.030 (4)	−0.070 (5)
C15	0.055 (3)	0.094 (4)	0.064 (3)	−0.012 (3)	−0.008 (2)	−0.019 (3)
C16	0.117 (5)	0.156 (7)	0.081 (4)	−0.052 (5)	−0.023 (4)	−0.035 (4)
C17	0.103 (5)	0.141 (7)	0.118 (6)	0.040 (5)	−0.030 (4)	−0.028 (5)
C18	0.100 (4)	0.074 (3)	0.058 (3)	0.002 (3)	−0.021 (3)	−0.017 (3)
C19	0.097 (4)	0.121 (5)	0.101 (5)	−0.013 (4)	−0.029 (4)	−0.048 (4)
Cl1	0.1360 (13)	0.1014 (11)	0.0782 (9)	−0.0581 (10)	0.0188 (9)	−0.0174 (8)
Cl2	0.1549 (15)	0.0523 (8)	0.1206 (13)	−0.0019 (9)	−0.0277 (11)	−0.0282 (8)
N1	0.058 (2)	0.046 (2)	0.055 (2)	−0.0101 (17)	0.0052 (17)	−0.0250 (18)
O1	0.106 (3)	0.061 (2)	0.068 (2)	−0.0291 (19)	0.0276 (19)	−0.0314 (17)
Sn1	0.0865 (3)	0.0497 (2)	0.0515 (2)	−0.01062 (16)	−0.00813 (16)	−0.01462 (15)

### Geometric parameters (Å, °)

**Table:**

C1—O1	1.264 (5)	C13—H13A	0.96
C1—C2	1.382 (6)	C13—H13B	0.96
C1—C4	1.502 (6)	C13—H13C	0.96
C2—C3	1.362 (5)	C14—H14A	0.96
C2—H2	0.93	C14—H14B	0.96
C3—N1	1.322 (5)	C14—H14C	0.96
C3—C5	1.494 (6)	C15—C17	1.506 (8)
C4—H4A	0.96	C15—C16	1.524 (8)
C4—H4B	0.96	C15—H15	0.98
C4—H4C	0.96	C16—H16A	0.96
C5—H5A	0.96	C16—H16B	0.96
C5—H5B	0.96	C16—H16C	0.96
C5—H5C	0.96	C17—H17A	0.96
C6—C11	1.381 (6)	C17—H17B	0.96
C6—C7	1.392 (6)	C17—H17C	0.96
C6—N1	1.435 (5)	C18—Sn1	2.095 (5)
C7—C8	1.376 (6)	C18—H18A	0.96
C7—C12	1.519 (6)	C18—H18B	0.96
C8—C9	1.366 (6)	C18—H18C	0.96
C8—H8	0.93	C19—Sn1	2.105 (5)
C9—C10	1.356 (6)	C19—H19A	0.96
C9—H9	0.93	C19—H19B	0.96
C10—C11	1.389 (6)	C19—H19C	0.96
C10—H10	0.93	Cl1—Sn1	2.3478 (16)
C11—C15	1.520 (6)	Cl2—Sn1	2.4644 (17)
C12—C14	1.497 (7)	N1—H1	0.833 (18)
C12—C13	1.505 (7)	O1—Sn1	2.375 (3)
C12—H12	0.98		
			
O1—C1—C2	121.9 (4)	C12—C14—H14A	109.5
O1—C1—C4	118.8 (4)	C12—C14—H14B	109.5
C2—C1—C4	119.3 (4)	H14A—C14—H14B	109.5
C3—C2—C1	125.3 (4)	C12—C14—H14C	109.5
C3—C2—H2	117.3	H14A—C14—H14C	109.5
C1—C2—H2	117.3	H14B—C14—H14C	109.5
N1—C3—C2	122.7 (4)	C17—C15—C11	112.4 (5)
N1—C3—C5	117.4 (4)	C17—C15—C16	110.5 (5)
C2—C3—C5	119.9 (4)	C11—C15—C16	109.5 (4)
C1—C4—H4A	109.5	C17—C15—H15	108.1
C1—C4—H4B	109.5	C11—C15—H15	108.1
H4A—C4—H4B	109.5	C16—C15—H15	108.1
C1—C4—H4C	109.5	C15—C16—H16A	109.5
H4A—C4—H4C	109.5	C15—C16—H16B	109.5
H4B—C4—H4C	109.5	H16A—C16—H16B	109.5
C3—C5—H5A	109.5	C15—C16—H16C	109.5
C3—C5—H5B	109.5	H16A—C16—H16C	109.5
H5A—C5—H5B	109.5	H16B—C16—H16C	109.5
C3—C5—H5C	109.5	C15—C17—H17A	109.5
H5A—C5—H5C	109.5	C15—C17—H17B	109.5
H5B—C5—H5C	109.5	H17A—C17—H17B	109.5
C11—C6—C7	121.8 (4)	C15—C17—H17C	109.5
C11—C6—N1	118.9 (4)	H17A—C17—H17C	109.5
C7—C6—N1	119.2 (4)	H17B—C17—H17C	109.5
C8—C7—C6	117.9 (4)	Sn1—C18—H18A	109.5
C8—C7—C12	120.8 (4)	Sn1—C18—H18B	109.5
C6—C7—C12	121.3 (4)	H18A—C18—H18B	109.5
C9—C8—C7	121.0 (4)	Sn1—C18—H18C	109.5
C9—C8—H8	119.5	H18A—C18—H18C	109.5
C7—C8—H8	119.5	H18B—C18—H18C	109.5
C10—C9—C8	120.6 (4)	Sn1—C19—H19A	109.5
C10—C9—H9	119.7	Sn1—C19—H19B	109.5
C8—C9—H9	119.7	H19A—C19—H19B	109.5
C9—C10—C11	120.8 (4)	Sn1—C19—H19C	109.5
C9—C10—H10	119.6	H19A—C19—H19C	109.5
C11—C10—H10	119.6	H19B—C19—H19C	109.5
C6—C11—C10	117.9 (4)	C3—N1—C6	125.1 (3)
C6—C11—C15	122.0 (4)	C3—N1—H1	117 (3)
C10—C11—C15	120.1 (4)	C6—N1—H1	117 (3)
C14—C12—C13	111.3 (5)	C1—O1—Sn1	136.4 (3)
C14—C12—C7	109.7 (4)	C18—Sn1—C19	142.9 (3)
C13—C12—C7	113.5 (4)	C18—Sn1—Cl1	107.61 (16)
C14—C12—H12	107.3	C19—Sn1—Cl1	107.20 (18)
C13—C12—H12	107.3	C18—Sn1—O1	88.67 (17)
C7—C12—H12	107.3	C19—Sn1—O1	84.0 (2)
C12—C13—H13A	109.5	Cl1—Sn1—O1	81.74 (9)
C12—C13—H13B	109.5	C18—Sn1—Cl2	94.04 (15)
H13A—C13—H13B	109.5	C19—Sn1—Cl2	94.78 (18)
C12—C13—H13C	109.5	Cl1—Sn1—Cl2	95.82 (6)
H13A—C13—H13C	109.5	O1—Sn1—Cl2	176.81 (8)
H13B—C13—H13C	109.5		
			
O1—C1—C2—C3	−1.0 (7)	C8—C7—C12—C14	87.0 (6)
C4—C1—C2—C3	178.8 (5)	C6—C7—C12—C14	−91.4 (5)
C1—C2—C3—N1	0.4 (7)	C8—C7—C12—C13	−38.2 (6)
C1—C2—C3—C5	179.4 (4)	C6—C7—C12—C13	143.3 (5)
C11—C6—C7—C8	0.0 (6)	C6—C11—C15—C17	−140.8 (5)
N1—C6—C7—C8	179.3 (4)	C10—C11—C15—C17	41.3 (7)
C11—C6—C7—C12	178.5 (4)	C6—C11—C15—C16	96.0 (5)
N1—C6—C7—C12	−2.2 (6)	C10—C11—C15—C16	−81.8 (6)
C6—C7—C8—C9	−1.3 (6)	C2—C3—N1—C6	−179.2 (4)
C12—C7—C8—C9	−179.8 (4)	C5—C3—N1—C6	1.7 (6)
C7—C8—C9—C10	1.0 (7)	C11—C6—N1—C3	86.6 (5)
C8—C9—C10—C11	0.6 (7)	C7—C6—N1—C3	−92.8 (5)
C7—C6—C11—C10	1.6 (6)	C2—C1—O1—Sn1	−156.7 (3)
N1—C6—C11—C10	−177.7 (4)	C4—C1—O1—Sn1	23.6 (7)
C7—C6—C11—C15	−176.3 (4)	C1—O1—Sn1—C18	−68.9 (5)
N1—C6—C11—C15	4.4 (6)	C1—O1—Sn1—C19	74.7 (5)
C9—C10—C11—C6	−1.8 (7)	C1—O1—Sn1—Cl1	−176.9 (5)
C9—C10—C11—C15	176.1 (4)		

### Hydrogen-bond geometry (Å, °)

**Table:**

*D*—H···*A*	*D*—H	H···*A*	*D*···*A*	*D*—H···*A*
N1—H1···O1	0.83 (3)	2.03 (4)	2.662 (5)	133 (4)
C8^i^—H8^i^···Cl1	0.93	2.91	3.695 (4)	143
C17^ii^—H17B^ii^···Cl2	0.96	2.89	3.783 (6)	155
C19^iii^—H19C^iii^···Cl2	0.96	2.94	3.700 (6)	137

Symmetry codes: (i) −*x*+1, −*y*+1, −*z*+2; (ii) *x*+1, *y*−1, *z*; (iii) −*x*+2, −*y*+1, −*z*+1.
